# Neuroendoscopic Resection of Intraventricular Tumors: A Systematic Outcomes Analysis

**DOI:** 10.1155/2013/898753

**Published:** 2013-09-26

**Authors:** Sean M. Barber, Leonardo Rangel-Castilla, David Baskin

**Affiliations:** Houston Methodist Neurological Institute, Department of Neurological Surgery, Suite 944, 6560 Fannin Street, Houston, TX 77030, USA

## Abstract

*Introduction*. Though traditional microsurgical techniques are the gold standard for intraventricular tumor resection, the morbidity and invasiveness of microsurgical approaches to the ventricular system have galvanized interest in neuroendoscopic resection. We present a systematic review of the literature to provide a better understanding of the virtues and limitations of endoscopic tumor resection. *Materials and Methods.* 40 articles describing 668 endoscopic tumor resections were selected from the Pubmed database and reviewed. *Results*. Complete or near-complete resection was achieved in 75.0% of the patients. 9.9% of resected tumors recurred during the follow-up period, and procedure-related complications occurred in 20.8% of the procedures. Tumor size ≤ 2cm (*P* = 0.00146), the presence of a cystic tumor component (*P* < 0.0001), and the use of navigation or stereotactic tools during the procedure (*P* = 0.0003) were each independently associated with a greater likelihood of complete or near-complete tumor resection. Additionally, the complication rate was significantly higher for noncystic masses than for cystic ones (*P* < 0.0001). *Discussion*. Neuroendoscopic outcomes for intraventricular tumor resection are significantly better when performed on small, cystic tumors and when neural navigation or stereotaxy is used. *Conclusion*. Neuroendoscopic resection appears to be a safe and reliable treatment option for patients with intraventricular tumors of a particular morphology.

## 1. Introduction

Intraventricular tumors present a unique challenge for the neurosurgeon. Their deep location and proximity to eloquent neurovascular anatomy complicate surgical approach and resection [1]. Microsurgery remains the gold standard for the treatment of intraventricular tumors [1–4], but microsurgical approaches are not without limitations [5–12]. The desire for a less invasive but equally effective surgical approach to intraventricular pathology has directed the attention of many in the neurosurgical community towards neuroendoscopy. 

Neuroendoscopy was introduced in the early 1900s, adopted initially by Dandy [13] and others [14, 15] as a novel means of treating hydrocephalus [16], but the technique was overshadowed midcentury by the advent of the valved ventriculoperitoneal (VP) shunt [17, 18]. Years later, neuroendoscopy regained popularity due to improvements in optical technology and the introduction of the rigid and flexible neuroendoscopes [16, 19, 20]. Today, neuroendoscopic techniques have further evolved, and the spectrum of intracranial pathologies treatable by modern neuro-endoscopic means continues to expand.

Early reports have demonstrated endoscopic resection of intraventricular masses to be effective and safe [21, 22]. The large majority of data in the neurosurgical literature, however, originate from studies of endoscopic colloid cyst resection [11, 23, 24]. Data regarding endoscopic resection of other intraventricular tumors exist primarily in case reports and small series with insufficient sample size to draw meaningful conclusions. 

The goal of this report is to review the relevant literature describing the endoscopic resection of intraventricular masses as a whole, both cystic and solid, to provide a better understanding of this technique's virtues and limitations.

## 2. Materials and Methods

Pubmed literature searches were performed using search terms “(endoscop*) AND ventric*”, “(endoscop*) AND tumor”, “((neuro-endoscop*) OR neuroendoscop*) AND tumor”, and “(tumor) AND ventric*”. Additional articles were located via cross-referencing of articles discovered initially through Pubmed searches. Articles included in the study were required to originate from peer-reviewed, English language journals describing the attempted resection (e.g., biopsies and cyst fenestrations without attempted resection were excluded) of an intraventricular tumor (e.g., suprasellar neoplasms without intraventricular extension were excluded) by purely endoscopic means (e.g., “endoscope-assisted” microsurgical resections were excluded) through a single endoscope (“dual-port” resections were excluded). Care was taken to exclude any redundant patient data from the analysis, and five articles required exclusion from the study due to an inability to definitively distinguish study patients in these five articles from patients in other study articles by the same author. In these five cases, the earlier of the two conflicting publications was omitted. Selected articles were also required to report on one or more of the following variables: (1) estimated completeness of resection achieved, (2) radiographic recurrence rates, and/or (3) complications related to the procedure. Cases involving the use of stereotactic radiosurgery, chemotherapy, or other nonsurgical treatment adjuncts were included. Two hundred and twenty articles were reviewed, and 40 were selected based on the above criteria.

Data collected from these 40 studies included tumor type, location within the ventricular system, tumor size, the presence of hydrocephalus preoperatively, operative technique, success of endoscopic resection, rates of intraoperative hemorrhage, and other procedure-related complications, rates of tumor recurrence, and length of clinical and/or radiographic follow-up.

Estimates regarding the completeness of endoscopic resection were obtained most commonly by surgeon or observer recollection and self-report, but were also obtained through assessments of postoperative imaging studies and chart review in some cases. Complete endoscopic resection was defined as gross total resection of all visible tumor as confirmed by visual intraoperative assessment or by the absence of any visible tumor residual on postoperative contrast magnetic resonance imaging (MRI). Near-complete resection was defined as resection of all but a very small amount of tumor adherent to nearby tissues. Partial resection was defined by a considerable tumor remnant as assessed either intraoperatively or on postoperative contrast MRI.

Statistical analysis was performed using the Student *t*-test and chi-square analysis using Microsoft Excel and GraphPad Instat 3 software. If the sample size was insufficient for chi-square testing (*n* < 5), the Fisher exact text was used. A *P* value of 0.05 was considered statistically significant.

## 3. Results

### 3.1. Patients and Tumor Types

The entire patient population consisted of 668 patients with intraventricular tumors who underwent attempted endoscopic resection. The publication dates of the 40 articles ranged from 1994 to 2012, and the number of patients (*n*) in each article ranged from 1 to 90 patients (mean, 16 patients). Hydrocephalus was seen preoperatively in 296 of 352 patients (84.1%) for whom relevant data was reported.

Colloid cysts were the most frequently encountered tumor by far (*n* = 569, 85.2% of study patients) followed by hypothalamic hamartomas (*n* = 30, 4.5% of study patients), craniopharyngiomas (*n* = 8, 1.2% of study patients), and ependymomas (*n* = 7, 1.0% of study patients). In 14 patients (2.1% of study patients) from 3 articles, the histological tumor type was either unknown or not reported. Tumor diameter ranged from 0.5 to 4.5 cm in 274 tumors from series where tumor size was reported (mean diameter, 1.5 cm). The most common tumor location was the third ventricle (*n* = 572, 85.2% of reported locations). Patient information and tumor types are summarized in Tables 1 and 2, respectively.

### 3.2. Operative Technique

Various techniques for neuroendoscopic resection of intraventricular tumors have been described in detail elsewhere [2, 12, 16, 20, 25–35]. Individual techniques differed throughout the included studies between surgeons as well as variances in tumor morphology and patient anatomy.

All procedures were performed with the patient under general anesthesia in a supine position. The patient's head was most commonly placed on a soft headrest, except where neuronavigation or stereotaxy was used, in which case the patient's head was placed in a 3-point pin fixation device. Preoperative antibiotics were always administered, but prophylactic antiepileptics frequently were not. The average operative time was 107.5 minutes and the average hospital stay was 4.8 ± 2.9 days.

Ventricular access was most commonly attained through a right-sided approach (unless asymmetric left-sided ventriculomegaly was present, in which case a left-sided approach was preferred). In all cases of hypothalamic hamartoma resection, ventricular access was performed contralateral to the greatest extent of tumor mass. Incision was made over the intended ventricular access site and a standard burr hole was created. The burr hole was most commonly placed at some variant of Kocher's point, although slightly more lateral (5–7 cm lateral to midline) on occasion. [3, 11, 36] Several authors make note of the importance of beveling the burr hole into a conical shape to allow for a greater degree of scope manipulation and visualization during the procedure [11, 37]. In some cases, the burr hole was placed more anteriorly (e.g., 5 cm anterior to the coronal suture, *n* = 183 [25, 26, 30, 31, 38, 39]; or 1.5–3 cm above the orbital rim in cases where a supraorbital trajectory was used, (*n* = 8 [27, 40])) to allow for better visualization of more posteriorly located tumors. In two cases, ventricular access was obtained via a transcallosal approach [12], and in the case of two pineal masses [41], a subtorcular approach was used.

The dura is incised in cruciate fashion and coagulated, followed by ventricular puncture and the introduction of an endoscope. Often a small-diameter peel-away introducer sheath containing a navigation probe and/or small-diameter rigid endoscope is used for initial ventricular puncture, although some authors preferred to perform initial ventricular puncture with a ventricular needle or catheter, followed by the introduction of an endoscope into the needle or catheter tract [31, 33]. 

### 3.3. Instruments

After entry into the ventricle, the tumor is inspected and its relationship to the surrounding anatomy is assessed. In some cases, visualization required the use of a 30° rigid endoscope or flexible neuroendoscope. A larger diameter rigid endoscope with multiple working channels is then introduced, through which tumor manipulation, coagulation, and resection take place. In the case of 59 colloid cysts and a single ependymoma, flexible neuroendoscopes were used for the majority of the procedure [2, 42, 43]. 

Cystic tumors were frequently penetrated and gently aspirated, after which the cyst wall was coagulated and resected piecemeal or *en bloc* with forceps, scissors, and other tools. In several cases, an adjunctive endoscopic aspiration tool (CUSA (Tyco Healthcare Radionics, Burlington, MA, USA) (*n* = 2) [41], NICO Myriad aspirator (NICO Corporation, Indianapolis, IN, USA) (*n* = 9) [41, 44, 45], Micro ENP Ultrasonic Hand Piece (Scoring GmbH, Medizintechnik, Germany) (*n* = 1) [42], or the Suros device (Suros Surgical Systems, Inc., Indianapolis, IN) (*n* = 2) [46]) assisted with tumor debulking and removal. 

### 3.4. Navigation/Stereotaxy

Navigation and/or stereotactic localization tools were used in 266 procedures (45.1% of 581 procedures reporting such data) [12, 25–29, 31, 33–35, 38, 39, 42, 46–49]. In some cases, navigation and/or stereotactic tools were used only in those patients lacking ventriculomegaly on preoperative imaging, due to the enhanced difficulty associated with endoscopic visualization and maneuverability in the absence of hydrocephalus. A single author describes the intraventricular insufflation of saline in cases where small ventricles are encountered in attempts to improve operative success in this setting [28]. Data regarding the use of navigation or stereotactic tools is summarized in Table 1.

### 3.5. Completeness of Resection

**Figure 1:**
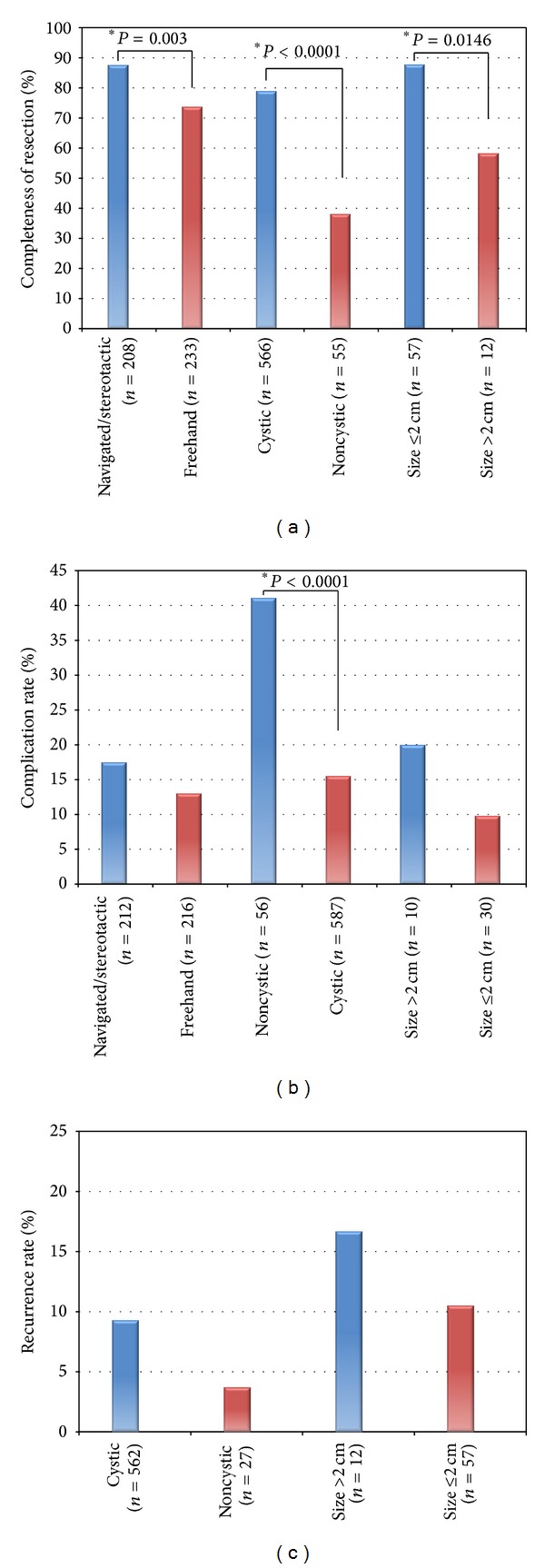
Column graphs displaying the variances in (a) resection success, (b) recurrence rate, and (c) complication rate seen with navigated endoscopic resection versus freehand, cystic tumors versus non-cystic, and large tumors (size > 2 cm) versus small (size ≤ 2 cm). *= statistically significant result.

Complete or near-complete tumor resection was achieved in 487 of 649 patients (75.0%) for whom completeness of endoscopic resection was reported. Complete resections were seen after initial resection attempts in 80.2% of colloid cysts, compared with 45.5% of other tumors (*P* < 0.0001). Complete or near-complete resection was more commonly attained amongst tumors with a substantial cystic component (79%) when compared with noncystic tumors (38.2%) (*P* < 0.0001). Complete or near-complete resection was also significantly more likely for tumors ≤2 cm in diameter when compared with larger tumors (*P* = 0.0146), and for tumors resected with the aid of navigation/stereotaxy (*P* = 0.0003) compared with those where these tools were not used. Resection outcomes are displayed in Figure 1 and Tables 1 and 2.

### 3.6. Adjunctive Procedures

Procedures in addition to the tumor resection were attempted during the same operative session in 70 patients (12.0% of patients for whom such data was reported). These adjunctive procedures included endoscopic third ventriculostomy (*n* = 27) [12, 16, 19, 29, 30, 42, 49, 50], septum pellucidostomy (*n* = 28) [12, 36, 49, 51], stent placement within the foramen of Monro and/or aqueduct of Sylvius (*n* = 2) [12, 19], placement of a VP-shunt [44] (*n* = 2), and postresection fluorescent ventriculography (*n* = 11) [34]. 

### 3.7. Procedure-Related Complications

Perioperative complications were seen in 123 out of 592 patients (20.8%) for whom data regarding complications was reported. These complications included hemorrhage (intraventricular, *n* = 41; intraparenchymal or along the introducer tract, *n* = 2; or epidural, *n* = 2), meningitis and/or ventriculitis (*n* = 15), “memory disturbance” (*n* = 14), CSF leak (*n* = 6), infarct (*n* = 5), cranial nerve deficit (*n* = 4), and hormonal disturbance (*n* = 2). The presence of a cystic component was associated with a significantly lower complication rate when compared to noncystic tumors (*P* < 0.0001). No significant relationship was observed between tumor size (*P* = 0.355) or the use of navigation/stereotaxy (*P* = 0.196) and complication rate. Data regarding procedure-related complications are shown in Figure 1 and Tables 1 and 2.

### 3.8. Clinical Outcomes

In the large majority of study patients, clinical morbidity was either unchanged or improved at most latent follow-up. There were no deaths reported to have occurred as a result of any of the 668 procedures. Postoperative morbidity increases were seen in 54 patients (9.5% of 569 patients for whom the relevant data was supplied) due to a variety of complications, including post-operative infarct, intraventricular hemorrhage, and meningitis or ventriculitis. Clinical outcomes are summarized in Table 1.

### 3.9. Tumor Recurrence

Tumor recurrence was seen in 53 of the 533 patients (9.9%) for whom data regarding recurrence was reported throughout an average of 31 months of follow-up. Recurrence was discovered, on average, 39 months after the initial resection in these 53 patients (range, 6–79 months). Tumor recurrence was seen in 9.8% of colloid cysts (49/498 patients reporting) compared with 11.1% of other tumors (4/36 patients reporting) (*P* = 0.805). Recurrence was seen most frequently with epidermoid cysts (*n* = 1, 100% recurrence), craniopharyngiomas (*n* = 5, 40% recurrence), and ependymomas (*n* = 1, 14.3% recurrence). No significant relationship was observed between tumor size (*P* = 0.546) or the presence of a cystic component (*P* = 0.325) and recurrence rates. Data regarding tumor recurrence are seen in Figure 1 and Tables 1 and 2.

## 4. Discussion

### 4.1. Virtues of Neuroendoscopic Tumor Resection

Neuro-endoscopy offers solutions to some of the challenges faced with intraventricular tumor surgery. Endoscopic approaches to intraventricular pathology provide improved illumination and visualization of an anatomically remote and otherwise-difficult-to-reach location without the degree of tissue dissection and retraction often required with microsurgical techniques [24, 52]. Early results taken from colloid cyst resection demonstrate a reduction in complication rates, overall morbidity, operative time, and hospital stay [20–22, 25]. 

Neuroendoscopic approaches to intraventricular pathology also afford the surgeon an opportunity to treat associated hydrocephalus concomitantly, although tumor resection alone may be sufficient to restore cerebrospinal fluid (CSF) flow in some cases [12, 24, 53, 54]. In our study, hydrocephalus was seen on presentation in 84.1% of intraventricular tumors undergoing endoscopic resection, yet adjunctive cerebrospinal fluid (CSF) diversionary procedures were performed along with tumor resection in only 12.0%.

### 4.2. Ideal Candidates for a Neuroendoscopic Approach

Neuroendoscopic resection appears to be most safe and effective [2, 21, 25, 34] when applied in a particular patient population and morphology of tumor. It is often suggested that small tumors, for example, are ideal candidates for neuroendoscopic resection [12, 23, 24, 32, 52]. Soft and/or cystic tumors are also preferred, as they lend themselves to rapid debulking via aspiration and/or other endoscopic techniques [12, 32]. Rigid tumors, in contrast, must be dissected and removed piecemeal with the fairly rudimentary tools available for endoscopic use. This may be too time-consuming of an endeavor to warrant the use of endoscopy in such cases. These principles appear substantiated by our findings that complete or near-complete resection was significantly more common for tumors with a large cystic component and those ≤2 cm in diameter.

 Neuroendoscopic resection is also best suited for relatively avascular tumors [23, 24], as endoscopic methods of acquiring timely hemostasis are lacking, and endoscopic visualization is largely compromised in the setting of active, uncontrolled hemorrhage [12, 32]. In our study, there was insufficient documentation of tumor vascularity within the included studies to draw meaningful conclusions about any relationship between tumor vascularity and variables such as resection success or complication rate. 

Ventriculomegaly is another factor which favors a neuroendoscopic approach. Small ventricles are thought to be unfavorable for neuroendoscopy because visibility and maneuverability in this setting are greatly reduced [12, 24, 63, 64], although several series provide evidence that endoscopic therapies are equally feasible in the absence of hydrocephalus [28, 65, 66]. 

### 4.3. Weaknesses of Neuroendoscopic Tumor Resection

Several of the limitations of neuroendoscopic tumor resection derive from a fundamental inadequacy of modern neuroendoscopic technology. As previously noted, solid masses greater than 2 cm in diameter, and those with considerable vascularity, are less amenable to neuroendoscopic resection due to the elementary nature of tools currently available for endoscopic dissection and hemostasis. 

The large majority of cases included in this study used forceps, suction catheters, and bipolar cautery as the primary tools for dissection, resection, and hemostasis, respectively. Several series, however, report on the use of assistive devices (e.g., CUSA, NICO Myriad aspirator, Micro ENP Ultrasonic Hand Piece, and the Suros device) designed to allow for rapid tumor dissection and removal through an endoscopic approach. Although surgeons who use these devices frequently report their being helpful, objective data regarding their overall benefit is lacking [42, 44, 45]. No significant difference in success of resection, complication rate, or clinical outcome was seen in our study with the use of these assistive devices, although their use was likely too infrequent (*n* = 8) to draw conclusions. 

Endoscopic tumor resections are also frequently said to result in inferior rates of gross total resection [25]. The resection rates demonstrated in our study (75.0%) and others (71–100%) [12, 32, 37, 65], however, appear comparable to those reported for microsurgical resection (80.4%–96%), particularly when endoscopic resection attempts are limited to tumors ≤2 cm in diameter (in which case resection rates in our analysis improve to 87.8%) [2, 67]. 

Some apprehension about the use of endoscopy for tumor resection arises from the perception that tumors resected endoscopically are more likely to recur [12, 21]. There is, in fact, some evidence that the risk of postoperative colloid cyst recurrence is higher with endoscopic resections compared with microsurgery [48]. Other series, however, have shown recurrence rates to be equivalent between the two [2]. The recurrence rate of 9.9% seen in our study is similar to rates reported for microsurgical resections (0.0%–33%) [32, 68–75], although reported recurrence rates vary widely and depend greatly on such variables as tumor type, completeness of initial resection, and the use of adjuvant therapies.

### 4.4. Stereotactic Tools and Neuronavigation

The use of stereotactic and/or neuronavigational guidance for endoscopic tumor resection is commonly reported in the neurosurgical literature, particularly in cases where ventriculomegaly is absent [12, 33, 65, 66, 76–78]. Some have adopted these adjunctive tools for assistance with burrhole placement, ventricular cannulation, and intraventricular navigation with the expectation that they will simplify the procedure and perhaps improve radiographic and clinical outcomes. Although incorporation of these tools into the procedure may prolong operative time and/or inflate surgical costs, several authors have declared their use to be of substantial benefit [12, 77–79]. Neuronavigation and/or stereotactic techniques were used in 44.1% of the cases in our study, and their use was associated with a significantly higher rate of complete or near-complete tumor resection. 

### 4.5. Complications

The overall complication rate of 20.8% seen in this study is consistent with values reported elsewhere for endoscopic resection (0–25%) [12, 28, 32, 35, 48, 76] and comparable to rates reported for microsurgical interventions (4.3–29.3%) [72, 80–84], although some reports of complications following microsurgical resection approach 70% [5, 11]. The complications seen most commonly in our study were intraventricular hemorrhage (which was frequently minor) and memory disturbance (which was often transient). Many of the complications observed did not translate into increased clinical morbidity, and most of the complication-related clinical morbidity resolved to some degree with time.

### 4.6. Study Limitations

We present the largest analysis to date of outcomes for endoscopic resection of intraventricular tumors. Limitations of this study include the following: (1) all included publications are retrospective and therefore subject to errors of confounding and bias. A more accurate comparison between surgical and endoscopic resection requires a prospective, randomized trial. (2) Data in our study is collected over an extended period of time. Being that endoscopic techniques have progressed appreciably over the last 25 years, our results may not provide an accurate assessment of the results attainable with modern techniques. A minor percentage of the data included in the study draws from resections utilizing flexible endoscopes, for example. Although some authors are proficient with flexible neuroendoscopes and have reported good outcomes with their use, modern rigid endoscopes offer a vastly improved image quality and are preferred by many neurosurgeons. (3) Available data in the literature draws largely from series of endoscopic colloid cyst resection and thus, represent a slightly skewed picture of endoscopic tumor resection. More data are needed regarding endoscopic resection of other tumor histologies if we hope to gain a truly accurate and complete understanding of the advantages and disadvantages of this technique. (4) Finally, the large majority of cases of endoscopic resection of intraventricular tumors in the literature describe tumors in the region of the third ventricle. The majority of intraventricular tumors, however, are discovered in the body or frontal horn of the lateral ventricle, followed by the atrium, and finally, the foramen of Monro and third ventricle [80, 81, 85]. More data may be needed regarding endoscopic resection of tumors in these more common locations before comments regarding the safety, efficacy, and overall usefulness of endoscopy in the treatment of intraventricular masses can be made.

## 5. Conclusion

The goal of this study was to better characterize the advantages and disadvantages of the endoscopic approach to intraventricular tumors. Our results indicate that endoscopic tumor resection, when applied in the appropriate setting, is safe and effective. 

Further improvements in the outcomes of neuroendoscopic tumor resection rely heavily on the development of endoscopic technology. Dissection tools allowing for the rapid and safe removal of large, solid tumors are lacking, as are effective means of acquiring prompt hemostasis through an endoscopic approach. More data is needed on the outcomes of endoscopic resection of tumors other than colloid cysts. Finally, randomized trials comparing surgical and endoscopic tumor resections would provide a better characterization of the virtues and limitations of each technique.

Microsurgical resection remains the gold standard of intraventricular tumor resection [1–4]. Endoscopic tools and techniques are improving, however, and the applications of endoscopy in the treatment of CNS pathology continue to expand. Though initial results appear promising, the potential of neuroendoscopy and its role in the management of intraventricular tumors are yet to be defined.

## Figures and Tables

**Table 1:** demonstrating articles included in the study by publication year with corresponding data regarding tumor histology, number of patients (*n*), presence of preoperative hydrocephalus, use of navigation/stereotactic tools, adjunctive endoscopic procedures, time spent in the operating room (OR time), hospital stay, procedure-related complications, resection success, and recurrence rate for 668 intraventricular tumors in 40 studies that underwent attempted endoscopic resection.

Author	Year	*n*	Tumor histology (*n*)	Preoperative Hydrocephalus (*n*)	Navigation/ stereotaxy (*n*)	Adjunctive procedures (*n*)	Mean OR time (min)	Mean hospital stay (days)	Complications (*n*)	Complete or near-complete resection (*n*) (%)	Recurrence (*n*)
Lewis et al. [11]	1994	7	Colloid cyst (7)	5	0	No	127	1.7	1	7 (100)	1
Abdullah and Caemaert [21]	1995	3	Craniopharyngioma (3)	ND	ND	ND	ND	ND	0	1 (33.4)	2
Abdou and Cohen [3]	1998	13	Colloid cyst (13)	13	0	No	ND	ND	0	10 (76.9)	0
Gaab and Schroeder [12]	1998	19	Colloid cyst (7), Subependymoma (3), low-grade astrocytoma (2), germinoma (1), pineal cyst (1), epidermoid cyst (1), hemangioma (1), cavernoma (1), CPP (1), ependymoma (1)	11	Navigation (4)	ETV (2), septostomy (1), stent (2)	85	ND	3	13 (68.4)	1
King et al. [51]	1999	13	Colloid cyst (13)	12	0	Septostomy (13)	94	2.3	2	10 (83.3)	0
Rodziewicz et al. [36]	2000	12	Colloid cyst (12)	6	0	Septostomy (12)	ND	ND	1	11 (91.7)	1
Decq et al. [55]	2000	22	Colloid cyst (22)	21	0	No	ND	ND	0	14 (63.6)	1
Kehler et al. [8]	2001	10	Colloid cyst (10)	ND	ND	ND	ND	ND	3	9 (90)	1
MacArthur et al. [56]	2002	7	Colloid cyst (3), low-grade astrocytoma (1), ependymoma (1), unknown (2)	ND	ND	ND	ND	ND	ND	4 (57.1)	0
Jho and Alfieri [57]	2002	2	Colloid cyst (2)	ND	0	No	ND	ND	0	2 (100)	0
Sgaramela et al. [58]	2003	1	Colloid cyst (1)	ND	ND	ND	ND	ND	0	1 (100)	0
Hellwig et al. [34]	2003	20	Colloid cyst (20)	19	Stereotaxy (9), Navigation (11)	Intraoperative ventriculography (11)	250 (stereotactic), 150 (navigated)	7	4	18 (90)	1
Husain et al. [20]	2003	25	Colloid cyst (11), ependymal cyst (2), choroid plexus cyst (2), septum Pellucidum cyst (2), arachnoid cyst (2), neurocysticercosis (2), craniopharyngioma (2), pineoblastoma (1), pineal Cyst (1)	ND	0	No	ND	3	2	20 (80)	ND
Souweidane [33, 37]	2005	2	Colloid cyst (1), glioneuronal tumor (1)	1	0	No	ND	ND	0	2 (100)	0
Jeon et al. [59]	2005	1	Choroid plexus cyst (1)	1	0	No	ND	37	1	1 (100)	ND
Longatti et al. [43]	2006	61	Colloid cysts (61)	53	0	No	87	6.7	4	38 (62.3)	7
Souweidane and Luther [32]	2006	7	Ependymoma (2), central neurocytoma (2), low-grade glioneuronal tumor (2), subependymoma (1)	7	0	No	117	2.6	2	5 (71.4)	0
Harter et al. [60]	2006	1	Dysembryoplastic neuroepithelial tumor (1)	1	ND	ND	ND	ND	ND	1 (100)	ND
Lekovic et al. [46]	2006	2	Hypothalamic hamartomas (2)	ND	Navigation (2)	No	ND	ND	0	1 (50)	ND
Grondin et al. [2]	2007	25	Colloid cysts (25)	22	0	No	104	3.8	3	24 (96)	1
Horn et al. [48]	2007	28	Colloid cysts (28)	17	Navigation (28)	No	174	5.4	3	10 (52.6), ND × 9	0
Levine et al. [61]	2007	35	Colloid cysts (35)	ND	ND	ND	ND	ND	7	32 (91.4)	7
Greenlee et al. [31]	2008	35	Colloid cysts (35)	ND	Frameless stereotaxy (35)	No	93	3	3	29 (82.8)	1
El-Ghandour [30]	2009	10	Colloid cysts (10)	10	0	ETV (2)	ND	ND	1	8 (80)	0
Stark et al. [38]	2009	1	Papillary ependymoma (1)	1	Navigation (1)	No	ND	ND	1	1 (100)	0
Romano et al. [50]	2009	1	Central neurocytoma (1)	1	0	ETV (1)	ND	ND	0	1 (100)	0
Oertel et al. [19]	2009	11	Unidentified (11)	ND	0	ETV (11)	71	ND	9	4 (36.3)	ND
Mishra et al. [39]	2010	59	Colloid cyst (59)	59	Navigation (59)	No	ND	ND	19	53 (89.8)	0
Najjar et al. [49]	2010	7	Colloid cyst (3), craniopharyngioma (1), low-grade astrocytoma (1), pineal cyst (1), unknown (1)	6	Navigation (2)	ETV (1), Septostomy (2)	ND	ND	0	4 (57.1)	1
Boogaarts et al. [29]	2011	90	Colloid cyst (90)	ND	Stereotaxy (18)	ETV (7)	79	ND	32	46 (57.5), ND × 10	24
Ahmad and Sandberg [16]	2010	1	CPP (1)	1	0	ETV (1)	ND	ND	0	1 (100)	0
Naftel et al. [28]	2011	4	Colloid cyst (2), hypothalamic hamartoma (2)	1	Navigation (2)	No	ND	ND	0	3 (75)	ND
Dlouhy et al. [45]	2011	4	Colloid cyst (3), pineoblastoma (1)	ND	ND	ND	ND	ND	ND	4 (100)	ND
Delitala et al. [27]	2011	7	Colloid cyst (7)	4	Navigation (4)	No	ND	ND	0	6 (85.7)	0
Sood et al. [41]	2011	2	Pineal cyst (1), Pineoblastoma (1)	2	0	No	ND	ND	ND	2 (100)	ND
Wilson et al. [26]	2012	22	Colloid cyst (22)	19	Navigation (19)	No	180	ND	0	21 (95.4)	0
Margetis and Souweidane [25]	2012	67	Colloid cyst (67)	ND	Navigation (67)	No	ND	ND	4	66 (98.5)	3
Mohanty et al. [44]	2012	3	Craniopharyngioma (2), subependymoma (1)	2	0	VP-shunt placement (2)	ND	ND	2	2 (66.7)	0
Selvanathan et al. [42]	2013	1	Ependymoma (1)	1	Navigation	ETV	ND	ND	1	1 (100)	1
Drees et al. [62]	2012	26	Hypothalamic hamartoma (26)	ND	ND	ND	ND	ND	14	0 (0)	ND

Total: 40 studies	Total: 668 patients			Total: 296/352 patients (84.1%)	Total: 262/581 patients (45.1%)	Total: 70/581 patients (12.0%)	Mean: 107.5 minutes	Mean: 4.8 days	Total: 123/592 patients (20.8%)	Total: 487/649 patients (75.0%)	Total: 53/533 patients (9.9%)

CPP: choroid plexus papilloma, ND: no data, ETV: endoscopic third ventriculostomy, VP-shunt: ventriculoperitoneal shunt, and min: minutes.

**Table 2:** displaying the various tumor histologies included in the study with corresponding data regarding the number of studies included, the number of patients, resection success, complication rates, and recurrence rates for each tumor type.

Tumor histology	Studied included (*n*)	Patients (*n*)	Complete or near-complete resection (*n*) (%)	Complications (*n*)	Recurrence (*n*)
Colloid Cyst	21	569	441/550 patients (80.2%)	83/556 patients (14.9%)	49/498 patients (9.8%)
Hypothalamic hamartoma	3	30	2/30 patients (6.7%)	14/30 patients (46.7%)	ND
Unidentified	3	14	6/14 patients (42.8%)	9/12 patients (75%)	0/3 patients (0%)
Craniopharyngioma	4	8	4/8 patients (50%)	1/8 patients (12.5%)	2/5 patients (40%)
Ependymoma	5	7	7/7 patients (100%)	4/6 patients (66.6%)	1/7 patients (14.3%)
Subependymoma	3	5	2/5 patients (40%)	2/5 patients (40%)	0/3 patients (0%)
Low-grade astrocytoma	3	4	1/4 patients (25%)	0/3 patients (0%)	0/4 patients (0%)
Pineal cyst	4	4	3/4 patients (75%)	0/3 patients (0%)	0/2 patients (0%)
Pineoblastoma	3	3	3/3 patients (100%)	0/2 patients (0%)	ND
Central neurocytoma	2	3	2/3 patients (33.4%)	0/3 patients (0%)	0/3 patients (0%)
Choroid plexus cyst	2	3	3/3 patients (100%)	1/3 patients (33.4%)	ND
Choroid plexus papilloma	2	2	2/2 patients (100%)	0/2 patients (0%)	0/2 patients (0%)
Septum pellucidum cyst	1	2	2/2 patients (100%)	0/2 patients (0%)	ND
Ependymal cyst	1	2	2/2 patients (100%)	0/2 patients (0%)	ND
Arachnoid Cyst	1	2	0/2 patients (0%)	0/2 patients (0%)	ND
Neurocysticercosis	1	2	1/2 patients (50%)	1/2 patients (50%)	ND
Neuroepithelial tumor	2	2	2/2 patients (100%)	0/1 patient (0%)	0/1 patient (0%)
Glioneuronal tumor	2	2	2/2 patients (100%)	0/2 patients (0%)	0/2 patients (0%)
Cavernoma	1	1	1/1 patient (100%)	1/1 patient (100%)	0/1 patient (0%)
Hemangioma	1	1	1/1 patient (100%)	0/1 patient (0%)	0/1 patient (0%)
Epidermoid cyst	1	1	0/1 patient (0%)	1/1 patient (100%)	1/1 patient (100%)
Germinoma	1	1	1/1 patient (100%)	1/1 patient (100%)	0/1 patient (0%)

ND: no data.
